# Anti-myeloma activity of MELK inhibitor OTS167: effects on drug-resistant myeloma cells and putative myeloma stem cell replenishment of malignant plasma cells

**DOI:** 10.1038/bcj.2016.71

**Published:** 2016-08-19

**Authors:** A T Stefka, J-H Park, Y Matsuo, S Chung, Y Nakamura, A J Jakubowiak, S Rosebeck

**Affiliations:** 1 Department of Medicine, University of Chicago, Chicago, IL, USA; 2 OncoTherapy Science, Inc., Kawasaki, Japan; 3 Department of Surgery, University of Chicago, Chicago, IL, USA

Currently considered incurable, multiple myeloma (MM) is characterized by proliferation of malignant plasma cells (PC) predominantly in the bone marrow, which overproduce monoclonal immunoglobulin proteins, and a perturbed tumor microenvironment, which promotes PC survival, inhibits osteoblast activity, increases osteoclast activity, and leads to hallmark osteolytic bone disease, all of which contribute to the clinical manifestations of the disease. Disrupting these sequelae by incorporation of proteasome inhibitors and immunomodulatory drugs into treatment regimens has improved overall survival of MM patients; however, the majority of patients will become refractory to the most effective currently available therapeutic options and relapse.^1^ It is hypothesized that a drug-resistant population of myeloma stem cells promotes disease relapse, but the characteristics and identity of this cell type(s) remain uncertain.^2^ Expression of maternal embryonic leucine zipper kinase (MELK) is increased in a number of cancers and is associated with poorer prognosis. MELK activity modulates many cellular and biological processes, including proliferation, apoptosis, hematopoiesis and oncogenesis, and is believed to have a critical role in cancer stem cell maintenance.^3^

We assessed the expression of *MELK* mRNA in malignant PC derived from MM patients and human myeloma cell lines (HMCL) and effects of the MELK inhibitor OTS167 on myeloma cells, including drug-resistant subclones. The effects of OTS167 were also tested in an *in vitro* cell culture model that recapitulates the bone marrow microenvironment and a malignant PC outgrowth model using peripheral blood mononuclear cells (PBMC) from patients with frank MM.

*MELK* gene expression analysis was performed on publically available data sets GSE5900,^4^ GSE2658 (refs 5, 6) and GSE6477 (ref. 7) and demonstrated significantly increased *MELK* mRNA expression in newly diagnosed MM PC (*n*=628) compared with either normal PC (nPC; *n*=37; *P*=0.0189), monoclonal gammopathy of undetermined significance (MGUS) CD138+ PC (*n*=65; *P*<0.0001), or PC from smoldering MM patients (*n*=36; *P*=0.0003; Figure 1a). There were no significant differences in *MELK* expression between nPC and either MGUS or sMM PC. Protein and mRNA expression of MELK were investigated in a panel of 26 patients from whom CD138+ MM PC were derived as well as 11 HMCL. Overall, MELK levels were variable with limited concordance between mRNA and protein (Supplementary Figures 1A and B).

Next, we tested the anti-myeloma effects of a potent, small-molecule inhibitor of MELK kinase activity, OTS167.^8^ Treatment decreased cell viability in a dose-dependent manner across 11 HMCL, which included dexamethasone-resistant MM1R, doxorubicin/bortezomib/carfilzomib cross-resistant 8226 Dox40 cells and carfilzomib-resistant KMS-34 CFZ cells (Figure 1b).^9, 10, 11, 12^ OTS167 treatment had comparable effects in both parental MM1S and KMS-34 and the resistant subclones MM1R and KMS-34 CFZ. Cross-resistant 8226 Dox40 cells, which overexpress the multidrug resistance channel ABCB1, were more resistant than parental 8226 and overall the most resistant to the effects of OTS167. Cellular IC_50_ values ranged from 5 to 60 nm.

To more specifically characterize the cytotoxic effects of MELK inhibition, we examined cell cycle distribution of p53 wild-type MM1S and p53 mutant U266 cells after 24 and 72 h of OTS167 treatment (Supplementary Figure 2A). With low-dose treatment, we observed arrest in G2/M phase of the cell cycle with a minor increase in sub-G1 DNA content in both cell lines, and high-dose treatment with OTS167 increased the proportion of cells with sub-G1 fragmented DNA. Western blot analysis of the same cells treated with OTS167 for 2–24 h showed rapid onset of PARP cleavage within the first 2–6 h of treatment (Figure 1c). Flow cytometric analysis of Annexin V/propidium iodide-stained MM1S and U266 cells treated for 24 h with MELK inhibitor demonstrated an increase in both early (Annexin V-positive/propidium iodide-negative) and late (Annexin V-positive/propidium iodide-positive) apoptotic cell populations (Supplementary Figure 2B). We also treated fresh bone marrow aspirates from two MM patients, one newly diagnosed and one with progressive disease, with OTS167 and analyzed apoptosis markers in either total marrow cells, CD138+ myeloma cells only, or other cells of the marrow not marked by CD138 expression (Figure 1d and Supplementary Figure 3). Although analysis of total marrow cells showed pre-existing levels of early apoptotic cells, the number of late apoptotic cells increased with MELK inhibition in a dose-dependent manner. Importantly, assessment of CD138+ PC showed 100% killing in response to OTS167, whereas the remaining CD138-negative cell population was markedly less affected by MELK inhibition.

Next, we treated MM1S and U266 cells with increasing doses of OTS167 for 24 h and analyzed expression of key myeloma survival proteins (Figure 1e). Expression of MELK and one of its known targets, FOXM1,^13^ as well as FOXM1 phosphorylation increased with the lowest doses and returned to lower levels at the higher doses, potentially as a result of enhanced cell death. Serine 473-phosphorylated Akt, which marks active Akt and was only detectable in MM1S cells, decreased in a dose-dependent manner and correlated with increased cleaved total Akt, which is associated with myeloma cell death. Myeloma survival factors c-Myc and IRF4 also decreased in a dose-dependent manner in both cell lines.^12^ p53 levels decreased in U266 cells but were only modestly changed in MM1S cells, and the p53 target, p21, was increased in p53 wild-type cells and fluctuated in p53 mutant cells, which agreed with previous findings.^14^ Altogether, these data reveal potent apoptotic effects of OTS167 in malignant MM PC, support the potential for OTS167 to overcome drug resistance, and define novel targets of MELK whose inhibition is known to promote myeloma cell death.

The bone marrow microenvironment potently influences the growth of MM cells and contributes to chemoresistance. This occurs, in part, via bone marrow stromal cell (BMSC)-dependent production of myeloma cell survival factors as well as direct interaction with MM PC.^1^ To test the potential of OTS167 to overcome growth-supportive effects conferred by BMSC, we co-cultured a subset of myeloma cell lines with the HS-5 BMSC line in the presence of increasing concentrations of OTS167 (Figure 2a). Both MM1S and RPMI 8226 cell lines demonstrated significant increases in overall cell growth when co-cultured with HS-5 BMSC, whereas multidrug-resistant 8226 Dox40 cells showed no significant additive growth effect. Importantly, treatment with OTS167-induced cell death in all three myeloma cell lines cultured alone and with BMSC, while having only a modest effect on the BMSC alone. Western blot analysis of replicate MM1S/HS-5 co-cultures demonstrates clear induction of apoptosis-associated PARP cleavage only in the presence of myeloma cells and not with HS-5 alone (Figure 2b). We also detected robust NF-κB activation, as determined by analysis of phospho-IκBα levels, in the HS-5 cells alone and in co-culture, and to a lesser extent in MM1S cells. NF-κB activation was impaired with increasing doses of OTS167. These results coupled with our flow cytometric analysis of enumerated total bone marrow samples underscore a potent anti-myeloma effect of MELK inhibition and establish OTS167 as a potential weapon for the treatment of chemoresistant myeloma.

Despite significant efforts, there is still no consensus for the identity and location of the myeloma stem cell population. It was demonstrated over 25 years ago that malignant MM precursor cells exist in the peripheral blood and can be induced to proliferate and differentiate into clonal MM PC with interleukin-3 (IL-3) and IL-6 treatment.^15^ Given the increasing evidence suggesting a role for MELK in cancer stem cells,^3^ we sought to determine the effects of OTS167 on the outgrowth and differentiation of presumptive myeloma stem cells into MM PC. Therefore we treated fresh MM patient PBMC cultures with IL-3/IL-6 alone or in combination with OTS167 for 6 days and then separated the resultant cells into CD138+ and CD138− populations using immunomagnetic positive selection (Figure 2c). IL-3/IL-6 treatment significantly increased the number of CD138+ cells and co-treatment with OTS167 impaired IL-3/IL-6-dependent growth of CD138+ cells. Interestingly, MELK inhibition alone reduced the number of CD138+ cells by fivefold. In the CD138− populations, no treatment significantly affected growth of the cells. This experiment, while indirect, demonstrates that MELK inhibition may kill a circulating MM progenitor cell population and/or prevent myeloma stem cells from re-establishing the malignant MM PC population.

In summary, this study establishes elevated *MELK* expression in symptomatic MM and implicates its potential in promoting myeloma cell growth and drug resistance. Importantly, MELK inhibition induced potent and rapid apoptosis of MM PC and impaired outgrowth of malignant PC derived from presumptive myeloma stem cells in the peripheral blood. These data, therefore, support clinical exploration into the potential of OTS167 as a new treatment option in MM with the ability to target active disease as well as insidious cells that may be responsible for disease relapse.

## Figures and Tables

**Figure 1:**
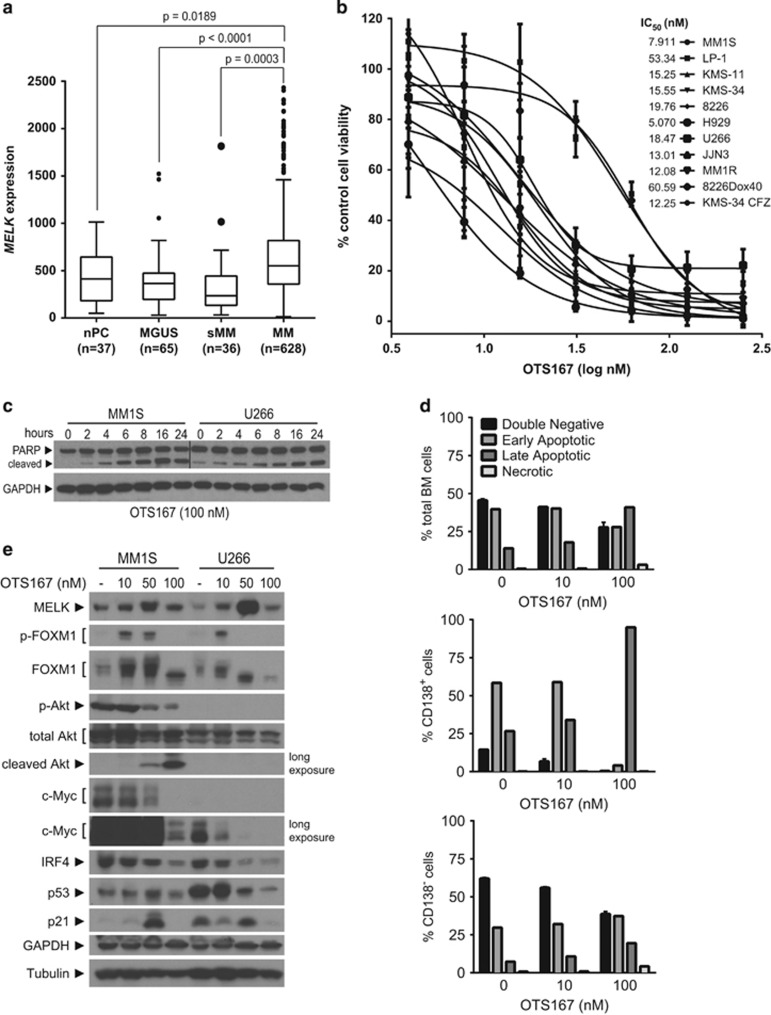
Expression and inhibition of MELK in MM cells. (**a**) Gene expression analysis of *MELK* mRNA expression was performed using publically available data sets, which include CD138+ PC from normal donors (nPC), monoclonal gammopathy of undetermined significance (MGUS), smoldering MM (sMM) or newly diagnosed MM. Statistical significance of differences (set at *P*<0.05) was determined using one-way analysis of variance with Tukey's multiple comparison test in GraphPad Prism 6 (GraphPad Software, San Diego, CA, USA). Adjusted *P*-values are indicated. (**b**) A panel of HMCL was exposed to increasing doses of OTS167 (OncoTherapy Science, Inc., Kawasaki, Japan) and cell viability was determined by MTT assay after 72 h. (inset) IC_50_ values were calculated in GraphPad Prism 6. (**c**) Western blot analysis of MM1S and U266 cells for PARP cleavage after treatment for 2–24 h with OTS167 at 100 nm. The vertical line indicates the merger of two scanned film images of different exposure times to depict comparable levels of PARP. (**d**) Total bone marrow samples were treated for 24 h with OTS167 at 10 and 100 nm, followed by staining with APC-CD138 (clone DL-101; BioLegend, San Diego, CA, USA) or control mouse IgG1 κ isotype (clone MOPC-21; BioLegend), Annexin fluorescein isothiocyanate and propidium iodide to monitor apoptosis in total bone marrow cells, CD138+ MM PC only, or cells of the marrow not marked by CD138 expression (CD138− cells). Data from representative experiments performed in duplicate are depicted. The percentage of live, early or late apoptotic and necrotic cells is shown. (**e**) MM1S and U266 cells were treated with increasing doses of OTS167 as indicated for 24 h before lysis and western blot analysis for the indicated proteins.

**Figure 2:**
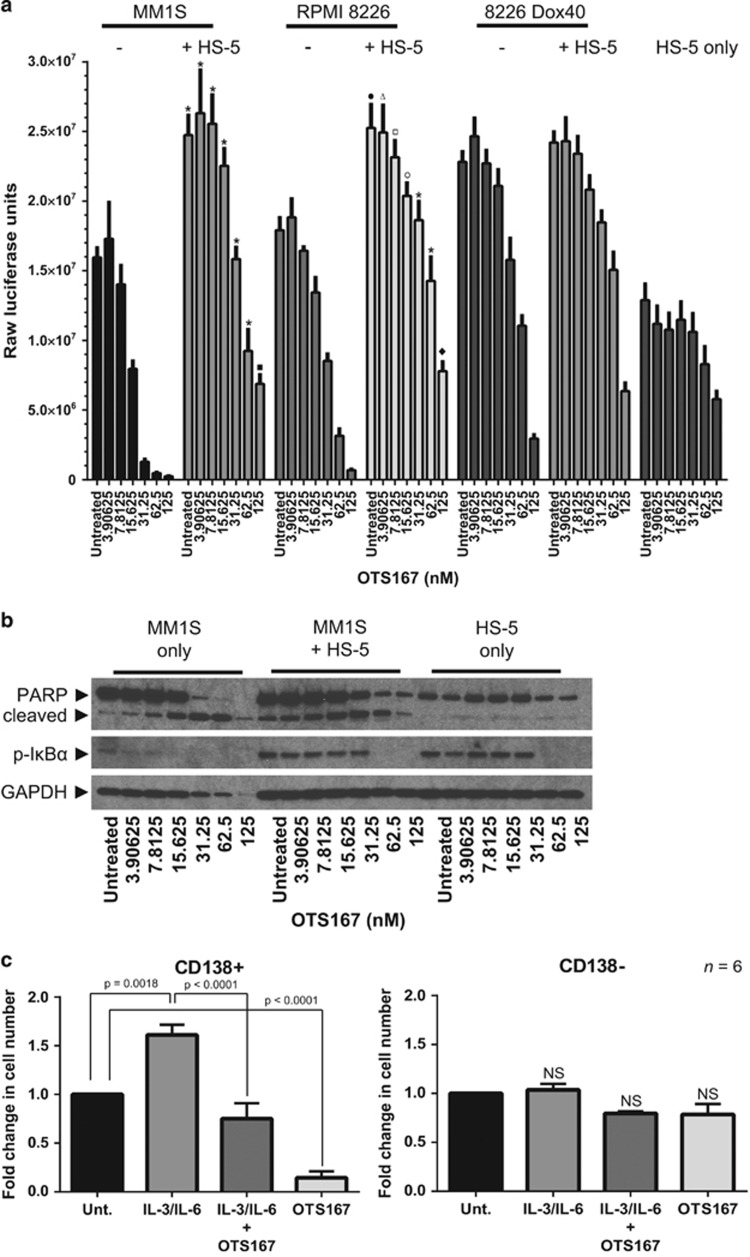
MELK inhibition overcomes drug resistance in the recapitulated bone marrow microenvironment and impairs putative myeloma stem cell repopulation of malignant MM PC. (**a**) The indicated cell lines were cultured alone or together as indicated and treated with OTS167 in increasing concentrations for 48 h before measuring metabolically active cell numbers using the CellTiter-Glo Luminescent Cell Viability Assay on a Glomax 96 microplate luminometer (Promega, Madison, WI, USA). Statistical significance of differences (set at *P*<0.05) was determined using two-way analysis of variance with Tukey's multiple comparison test in GraphPad Prism 6 (GraphPad Software, La Jolla, CA, USA). Adjusted *P*-values are indicated. Raw luciferase units are reported. Results shown are of two experiments performed in triplicate. *P<0.0001; ▪*P*=0.0063; •*P*=0.0015; Δ*P*=0.0167; □*P*=0.0053; ○*P*=0.0035; ♦ *P*=0.0024. (**b**) Western blot analysis of MM1S and HS-5 stromal cells cultured alone or together with increasing concentrations of OTS167 for 48 hours demonstrates preferential apoptosis in myeloma cells. (**c**) Peripheral blood mononuclear cells (PBMC) were obtained by Ficoll gradient separation and 2.0–2.5 × 10^6^ PBMC/well in six-well plates were cultured as indicated with recombinant human interleukin-3 (IL-3) and IL-6 (both at 5 ng/ml), purchased from R&D Systems (Minneapolis, MN, USA) (catalog number 203-IL and 206-IL, respectively), or OTS167 (10 nm) alone or in combination for 6 days. Cultures were collected and subjected to immunomagnetic positive selection to separate CD138+ plasma cells from CD138− cells. Trypan blue-negative cells were counted and the fold change in each population in response to each treatment is depicted. Data represent six independent determinations from six separate blood samples from MM patients. Statistical significance of differences (set at *P*<0.05) was determined using one-way analysis of variance with Tukey's multiple comparison test in GraphPad Prism 6 (GraphPad Software). *P*-values are indicated. Effects on CD138− populations are not significant (NS).
