# Supplementary material for: Anti-myeloma activity of MELK inhibitor OTS167: effects on drug-resistant myeloma cells and putative myeloma stem cell replenishment of malignant plasma cells
Source: Blood Cancer J. 2016 Aug 19;6(8):e460–. doi: 10.1038/bcj.2016.71 (PMC5022182; doi:10.1038/bcj.2016.71)
Supplement: Supplementary Information [file bcj201671x1.doc]

**Supplementary Information**

Supplementary information includes three figures and associated legends.

Supplementary Figures


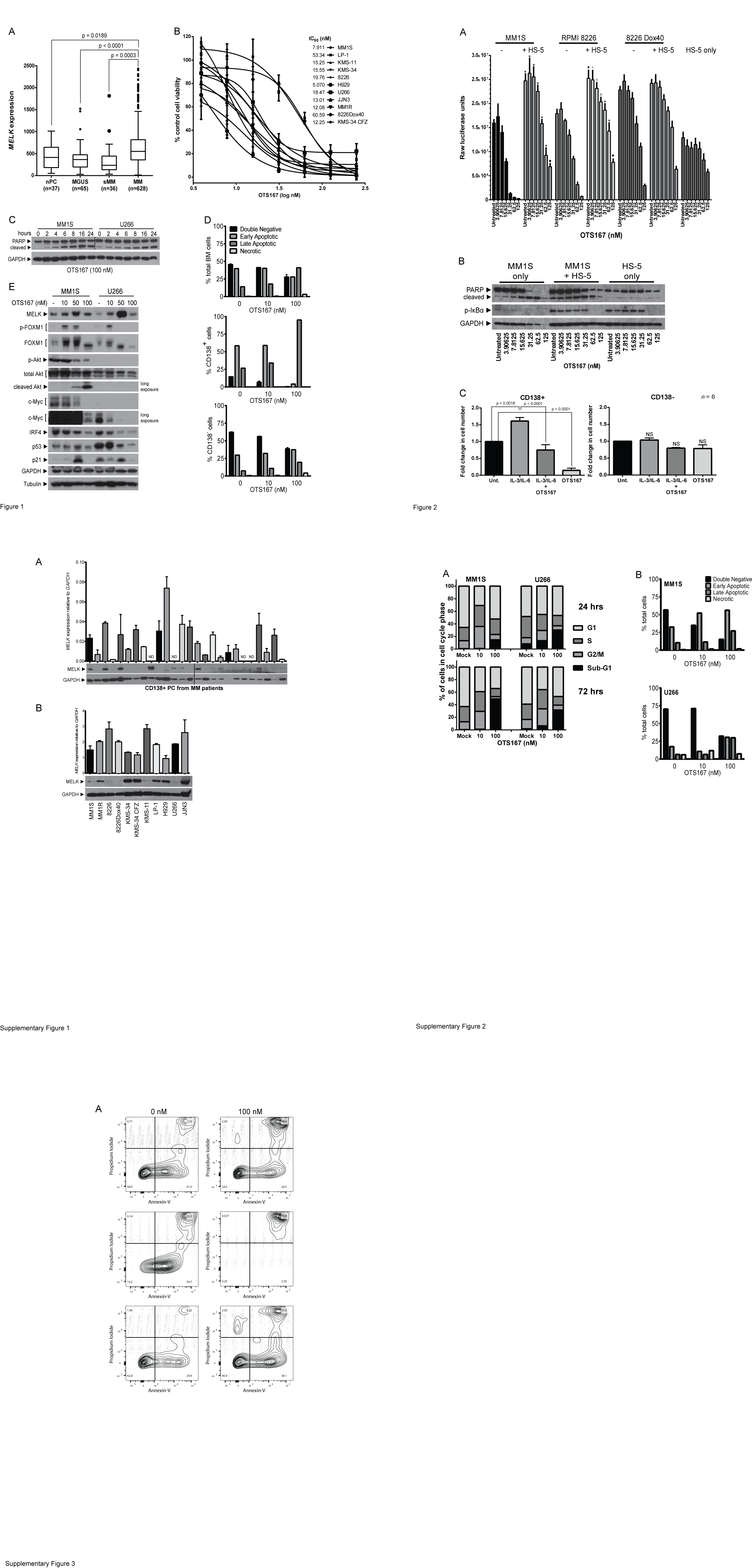


**Supplementary Figure 1.** **mRNA and protein expression of MELK in patient-derived CD138+ MM PC and myeloma cell lines.** *Upper graphs* - (A) Total RNA was isolated from CD138+ plasma cells derived from 26 MM patients using the AllPrep DNA/RNA Micro Kit (Qiagen) or (B) from HMCL using the RNeasy mini kit (Qiagen). First-strand cDNA synthesis was performed using the High Capacity cDNA Reverse Transcription Kit (Applied Biosystems). qPCR was carried out using previously described primer sets (Chung *et al* 2012) and SYBR green detection on a StepOnePlus Real-time PCR System (Applied Biosystems). Expression levels of *MELK* were calculated relative to *GAPDH* using the ΔC_T_ method. *Lower panels* - (A) Acetone-precipitated proteins from RNA column flow-through from CD138+ MM plasma cells or (B) lysates from HMCL were analyzed for expression of MELK protein. Antibodies for western blot included anti-MELK (MAB4331) from EMD Millipore and anti-GAPDH (sc-32233) from Santa Cruz Biotechnology. ND - not detected. The vertical line indicates separate scanned film images merged for the purpose of contiguous comparison.

**
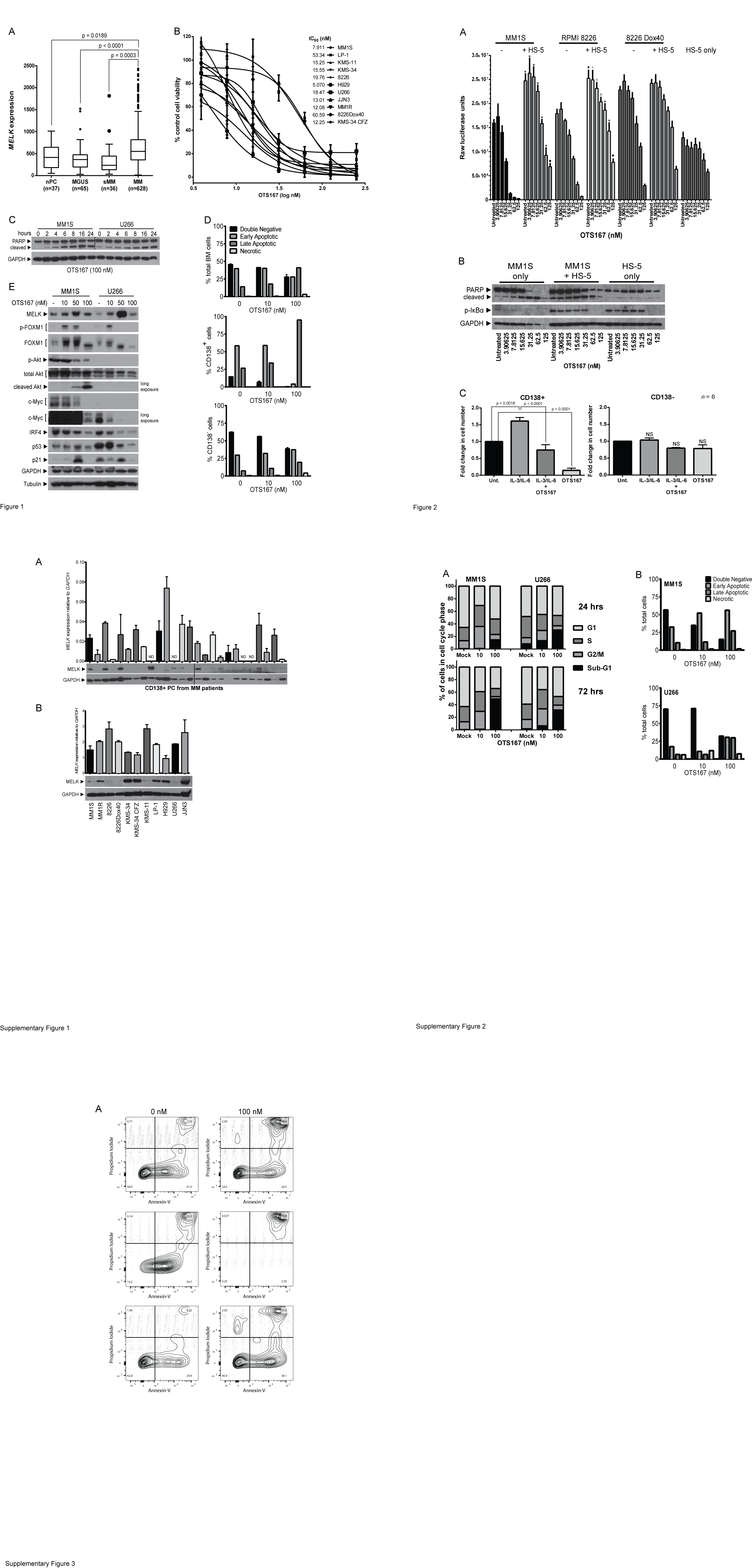
**

**Supplementary Figure 2. Cell cycle and apoptosis analysis of p53 wild-type MM1S and p53 mutant U266 cells.** (A) For cell cycle analysis, HMCL were treated for 24 and 72 hours with 10 and 100 nM OTS167, fixed, then stained with propidium iodide (PI). Distribution of cells within each phase of the cell cycle was determined by examination of DNA content by flow cytometry. (B) MM1S and U266 cells were treated for 24 hours with OTS167 at 10 and 100 nM and stained with Annexin V-FITC and PI to monitor the induction of apoptosis (Annexin V-FITC Early Apoptosis Detection Kit; #6592; Cell Signaling Technology).

**
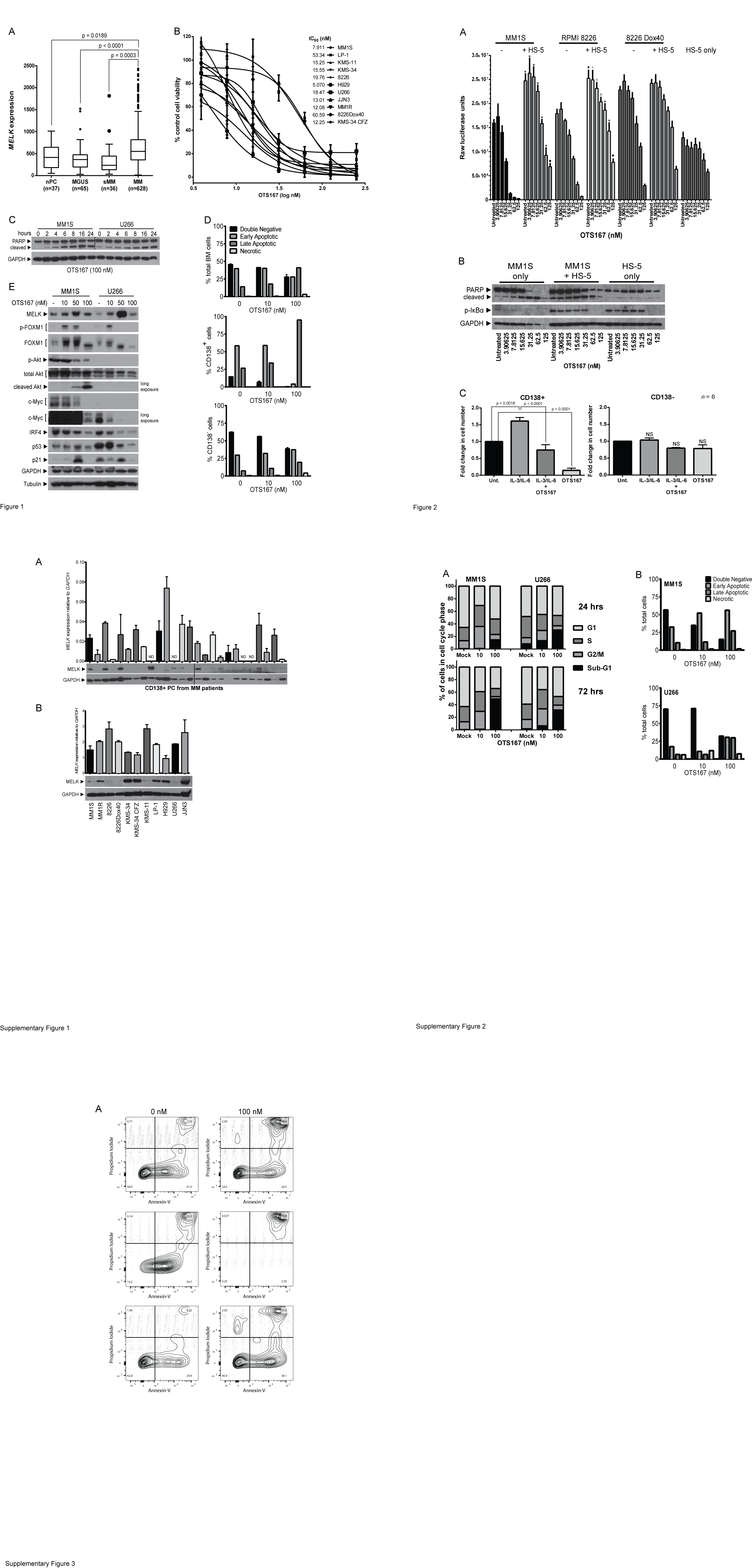
**

CD138-positive cells

CD138-negative cells

Total BM MNC

**Supplementary Figure 3.** **Representative density plots used in the determination of changes in Annexin V and PI.** Fresh patient-derived bone marrow mononuclear cells (BM MNC) were treated with OTS167 as indicated for 24 hours. Following treatment, cells were harvested and stained as previously described with Annexin V/PI and anti-CD138. Analysis of the indicated populations was performed to enumerate Annexin V/PI staining in total BM MNC, CD138+ only, and cells of the BM not marked by CD138.
